# Durability and Cross-Reactivity of SARS-CoV-2 mRNA Vaccine in Adolescent Children

**DOI:** 10.3390/vaccines10040492

**Published:** 2022-03-23

**Authors:** Madeleine D. Burns, Brittany P. Boribong, Yannic C. Bartsch, Maggie Loiselle, Kerri J. St. Denis, Maegan L. Sheehan, Jessica W. Chen, Jameson P. Davis, Rosiane Lima, Andrea G. Edlow, Alessio Fasano, Alejandro B. Balazs, Galit Alter, Lael M. Yonker

**Affiliations:** 1 Massachusetts General Hospital Department of Pediatrics, Mucosal Immunology and Biology Research Center, Boston, MA 02114, USA; mburns18@mgh.harvard.edu (M.D.B.); bboribong@mgh.harvard.edu (B.P.B.); mloiselle@mgh.harvard.edu (M.L.); jpdavis@mgh.harvard.edu (J.P.D.); rslima@mgh.harvard.edu (R.L.); afasano@mgh.harvard.edu (A.F.); 2 Ragon Institute of MGH, MIT, and Harvard, Cambridge, MA 02139, USA; ybartsch@mgh.harvard.edu (Y.C.B.); kstdenis@balazslab.com (K.J.S.D.); msheehan@balazslab.com (M.L.S.); jessicachen@g.harvard.edu (J.W.C.); abalazs@mgh.harvard.edu (A.B.B.); galter@mgh.harvard.edu (G.A.); 3 Massachusetts General Hospital Department of Obstetrics and Gynecology, Division of Maternal-Fetal Medicine, Vincent Center for Reproductive Biology, Boston, MA 02114, USA; aedlow@mgh.harvard.edu

**Keywords:** SARS-CoV-2, COVID-19, pediatrics, adolescents, vaccines, boosters, immunology, antibodies

## Abstract

Emergent SARS-CoV-2 variants and waning humoral immunity in vaccinated individuals have resulted in increased infections and hospitalizations. Children are not spared from infection nor complications of COVID-19, and the recent recommendation for boosters in individuals ages 12 years or older calls for broader understanding of the adolescent immune profile after mRNA vaccination. We tested the durability and cross-reactivity of anti-SARS-CoV-2 serologic responses over a six-month time course in vaccinated adolescents against the SARS-CoV-2 D614G (“wild type”) and Omicron antigens. Serum from 77 adolescents showed that anti-Spike antibodies wane significantly over six months. After completion of a two-vaccine series, cross-reactivity against Omicron-specific receptor-binding domain (RBD) was seen. Functional humoral activation against wild type and Omicron SARS-CoV-2 also declines over time in vaccinated adolescent children. Evidence of waning mRNA-induced vaccine immunity underscores vulnerabilities in long-term pediatric protection against SARS-CoV-2 infection, while cross-reactivity highlights the additional benefits of vaccination. Characterization of adolescent immune signatures post-vaccination will inform guidance on vaccine platforms and timelines, and ultimately optimize immunoprotection of children.

## 1. Introduction

As we entered the third year of the COVID-19 pandemic, SARS-CoV-2 infection rates surged due to highly infectious viral variants and waning population immunity. Despite full (two-dose) mRNA vaccination [1], there are still increasing infections and hospitalizations among those individuals who have been vaccinated. Thus, boosters are now recommended for adults five months after full vaccination. More recently, there have been recommendations for adolescents (ages 12–18 years old) to obtain boosters, yet little is known of the durability and long-term efficacy of the adolescent post-vaccination immune profile.

While mortality from COVID-19 is lower in children compared to adults, over 12.7 million children have been diagnosed with COVID-19, leading to roughly 40,000 hospitalizations in the US [2] and over 12,800 pediatric deaths worldwide as of March 2022 [3]. Additionally, over 7400 children in the US have been diagnosed with the post-infectious illness, Multisystem Inflammatory Syndrome in Children (MIS-C) [4], and more than one in seven children experience long COVID-19 [5]. Completion of a two-dose mRNA vaccine series is highly effective in preventing MIS-C, and children with MIS-C who are critically ill are more likely to be unvaccinated [6]. Children are not spared from infection, and vaccination remains a critical strategy for preventing infection and transmission, reducing severity of disease, and limiting complications of COVID-19 [7].

The emergence of the Omicron variant has called into question the long-term efficacy of current vaccine platforms and dosing regimens in children. Recent data now suggest that BNT16b2 vaccine-induced immune protection declines rapidly in children, especially children aged 5–11 years [8]. Children under 5 years of age still remain ineligible for a SARS-CoV-2 vaccine, and a third dose will likely be needed to reach appropriate protection for children ages 6 months through 4 years old [9]. Booster doses can restore vaccine efficacy in older adolescents [10], but with easing mask mandates, transmission and infection amongst vaccinated children remains possible. Immune responses from children across all pediatric age groups must be understood to optimize vaccination strategies with the goal of preventing infection and associated complications.

Here, we quantified relative antibody responses in adolescent children immediately following the Pfizer-BioNTech mRNA vaccination and six months post-inoculation and analyzed the efficacy of the humoral response against the D614G (“wild type”) SARS-CoV-2 and latest variant of concern (VOC), Omicron.

## 2. Materials and Methods

### 2.1. Study Design and Sample Collection

Adolescent children (ages 12–17) assented, with parental consent, to participate in the MGH Pediatric COVID-19 Biorepository (MGB IRB #2020P000955) [11]. Young adults ages 18–19 years of age provided their own consent to participate. Demographic information was extracted from medical records. Blood was collected into serum separation tubes by venipuncture or by microneedle device prior to vaccination (V0), 2–3 weeks after the initial Pfizer-BioNTech mRNA vaccination (V1), 2–4 weeks after the second dose (V2), and again 6 months after the vaccination series was complete (V6). Children were given the option to give a blood sample at any or all the time points. Blood was allowed to clot, then spun by centrifuge, and serum was collected. Samples were collected from May 2021–January 2022.

### 2.2. Enzyme-Linked Immunosorbent Assay (ELISA)

Serological analyses were performed using an in-house enzyme-linked immunosorbent assay (ELISA) that detects IgG against the D614G (“wild type”) SARS-CoV-2 Spike, the D614G (“wild type”) Receptor-Binding Domain (RBD), or the Omicron SARS-CoV-2 VOC RBD by using the previously described method [7]. Briefly, 96-well plates were coated with 1 µg/mL of Spike or RBD overnight at 4 °C in bi-carbonate buffer. The plates were then washed, and serum samples were added at a 1:500 (Spike) dilution or 1:200 (RBD) dilution in duplicate for 1 h at room temperature, washed, and then detected with a secondary anti-IgG (Bethyl Laboratories; Montgomery, TX, USA). The secondary was washed away after 1 h, and the colorimetric detector was added (TMB; Thermo Fisher Scientific; Waltham, MA, USA) for 5 min. The reaction was then stopped, and the absorbance was acquired at 450/570 nm on a SpectraMax plate reader. To convert raw OD values into concentration, a two-fold dilution curve (starting at 29.8 international units) of the WHO standard (NIBSC code: 20/136) was included onto every ELISA plate. The sample concentration was interpolated from the resulting standard curve, as previously described [12]. Antibody responses at each time point were analyzed relative to the average V0 (pre-vaccination) antibody response.

### 2.3. Virus Neutralization

The neutralizing activity of vaccine sera against coronaviruses was compared by producing lentiviral particles pseudotyped with different Spike proteins, as previously described [13]. Neutralization assays and readout were performed on a Fluent Automated Workstation (Tecan; Männedorf, Zürich Switzerland) liquid handler using 384-well plates (Grenier; Monroe, NC, USA). Three-fold serial dilutions were performed for each serum sample before adding 50–250 infectious units of pseudovirus expressing the SARS-CoV-2 wild type or Omicron variant Spike to hACE-2 expressing HEK293 for 1 h. Dilutions for sera ranged from 1:12 to 1:8748. Percent neutralization was determined by subtracting background luminescence measured in cell control wells (cells only) from sample wells and dividing by virus control wells (virus and cells only). Pseudovirus neutralization titers [pNT50] values were calculated by taking the inverse of the 50% inhibitory concentration value for all samples with a pseudovirus neutralization value of 80% or higher at the highest concentration of serum.

### 2.4. Antibody-Dependent-Complement-Deposition (ADCD)

Complement deposition was performed as previously described [14]. Briefly, biotinylated antigens were bound to FluoSphere NeutrAvidin beads (Thermo Fisher Scientific; Waltham, MA, USA). To form immune-complexes, antigen-coated beads were incubated with 10 µL of 1:10 diluted serum samples. Non-specific antibodies were washed away, and immune complexes were incubated with guinea pig complement in GVB++ buffer (Boston BioProducts; Milford, MA, USA). Complement reaction was stopped using EDTA containing PBS (15 mM). Deposited C3 on beads were stained with anti-guinea pig C3-FITC antibody (MP Biomedicals; Irvine, California USA, 1:100, polyclonal) and analyzed on an anti-guinea pig C3-FITC antibody (MP Biomedicals, CA, USA, 1:100, polyclonal) and analyzed on an iQue analyzer (Intellicyt; Albuquerque, NM, USA).

### 2.5. Antibody-Dependent-THP-1 Cellular-Phagocytosis (ADCP)

THP-1 cellular phagocytosis assay was performed as previously described [15]. Briefly, biotinylated antigens were bound to FluoSphere NeutrAvidin beads (Thermo Fisher Scientific; Waltham, MA, USA). To form immune-complexes, antigen-coated beads were incubated with 10 µL of 1:100 diluted serum samples. THP-1 monocytes were added to the bead mixture and incubated at 37 °C. After 16 h incubation, cells were fixed with 4% paraformaldehyde and analyzed on an iQue analyzer (Intellicyt; Albuquerque, NM, USA).

### 2.6. Antibody-Dependent-Neutrophil-Phagocytosis (ADNP)

Neutrophil phagocytosis assay was performed as previously described [16]. Briefly, biotinylated antigens were bound to FluoSphere NeutrAvidin beads (Thermo Fisher Scientific; Waltham, MA, USA). To form immune-complexes, antigen-coated beads were incubated with 10 µL of 1:50 diluted serum samples. Primary neutrophils were isolated from Ammonium-Chloride-Potassium (ACK) buffer-lysed whole blood from healthy controls. Neutrophils were incubated with washed immune complexes at 37 °C. After 1 h incubation, neutrophils were stained for surface marker CD66b (Biolegend; San Diego, CA, USA; 1:100, clone: G10F5), fixed with 4% paraformaldehyde, and analyzed on an iQue analyzer (Intellicyt; Albuquerque, NM, USA).

### 2.7. Statistical Analysis

Analysis was completed by Prism 9.3 using one-way ANOVA for multiple comparisons and *t*-test for two-way comparisons. Correlations were completed using Pearson correlation. Outliers were removed using a Robust regression and Outlier removal (ROUT) method with a Q value of 0.02%.

## 3. Results

Seventy-seven children were enrolled in our study, with an average age of 14 years; sixty-eight children were between the ages of 12–15 years; nine were between ages of 16-19 years. Sex was equally distributed, and 19% of the population was Hispanic (Table 1). Ninety-five percent (n = 73) of participants denied SARS-CoV-2 infection prior to enrollment and throughout the course of the study. Thirty-one percent (n = 24) of the children provided blood samples at all four separate time points: prior to vaccination (V0), 2–3 weeks after first vaccine dose (V1), 2–4 weeks after the second vaccine dose (V2), and 6 months after the second vaccine dose (V6).

**Figure 1:**
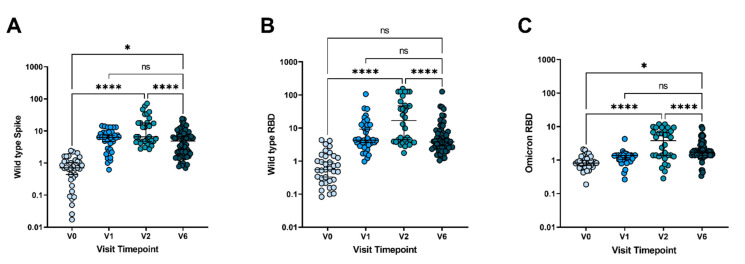
Adolescent anti-SARS-CoV-2 antibody responses over time. Relative humoral responses to (**A**) Wild type Spike (**B**) Wild type Receptor Binding Domain (RBD), and (**C**) Omicron RBD are quantified prior to vaccination, 2–3 weeks following the first vaccine dose, 2–4 weeks following the second mRNA vaccine dose, and 6 months following the second mRNA vaccine dose. V0 = pre-vaccination, V1 = 2–3 weeks following the first vaccine dose, V2 = 2–4 weeks following the second mRNA vaccine dose, and V6 = 6 months following the second mRNA vaccine dose. Displayed as fold increase from baseline. Analysis by ANOVA. ns = not significant, * *p* < 0.05, **** *p* < 0.0001. Median values and 95% CI are presented.

**Figure A1:**
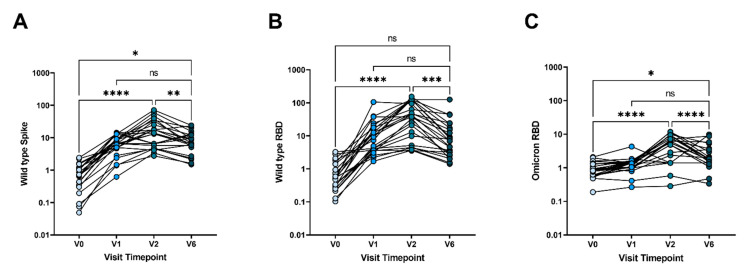
Individual anti-SARS-CoV-2 antibody responses over time. Twenty-four adolescents provided blood samples at each of the four time points. Relative humoral responses to (**A**) wild type Spike, (**B**) wild type Receptor Binding Domain (RBD), and (**C**) Omicron RBD were quantified prior to vaccination. V0 = pre-vaccination, V1 = 2–3 weeks following the first vaccine dose, V2 = 2–4 weeks following the second mRNA vaccine dose, and V6 = 6 months following the second mRNA vaccine dose. Displayed as fold increase from baseline. Analysis by ANOVA. ns = not significant, * *p* < 0.05, ** *p* < 0.01, *** *p* < 0.001 **** *p* < 0.0001.

Relative antibody responses to SARS-CoV-2, wild type Spike (Figure 1A), wild type RBD (Figure 1B), and Omicron RBD (Figure 1C) were analyzed for each time point. Robust generation of anti-wild type and anti-Omicron antibodies were seen following the second dose of the vaccine, as compared to pre-vaccination levels (*p* < 0.0001, *p* < 0.0001, *p* < 0.0001; wild type Spike, wild type RBD, and Omicron RBD, respectively). Interestingly, there was no increase in antibodies against Omicron RBD after the first vaccine dose, but a significant increase in titers was seen following a second vaccine dose. However, there was a subsequent loss of all anti-SARS-CoV-2 antibody responses by six months, as compared to the V2 time point (*p* < 0.0001, *p* < 0.0001, *p* < 0.0001; wild type Spike, wild type RBD, and Omicron RBD). By six months, antibody responses decreased to levels comparable to titers seen at the V1 time point, following the first vaccine dose. Twenty-four adolescents provided blood samples at all four time points; individual responses align with trends seen in the larger cohort (Figure A1). This loss of antibody titers highlights a potential vulnerability of adolescents and young adults to breakthrough infections six months after the completion of a two-dose vaccine series.

**Figure 2:**
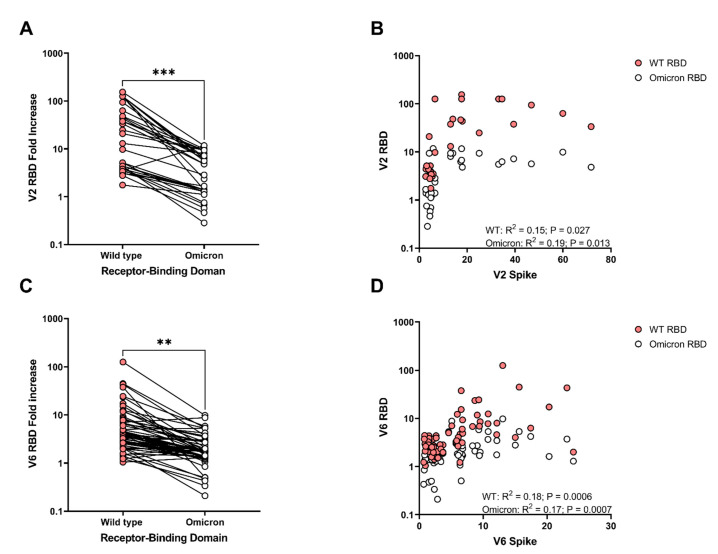
Comparison of humoral response to SARS-CoV-2 wild type and Omicron Receptor Binding Domain (RBD). (**A**) Following the second mRNA vaccine dose, anti-RBD responses titers are compared between wild type and Omicron, (**B**) and correlations between RBD for each variant and Spike were assessed. (**C**) Anti-RBD titers were also compared at the 6-month time point, (**D**) and the correlation between RBD and Spike was again assessed. V2 = 2–4 weeks following the second mRNA vaccine dose, and V6 = 6 months following the second mRNA vaccine dose. Paired analysis with t-test, correlation with Pearson correlation. WT = wild type. ** *p* < 0.01, *** *p* < 0.001.

As Omicron has become the predominant SARS-CoV-2 variant globally, we assessed the relationship between anti-wild type RBD and anti-Omicron RBD titers to determine if COVID-19 mRNA vaccination displayed cross-coverage and potential protection against the Omicron-specific SARS-CoV-2 RBD. While a single vaccination produces only low titers against Omicron, the second vaccination establishes greater cross-reactivity of RBD responses in many, but not all, adolescents (Figure 1C), and the titers remain significantly lower for Omicron RBD than for wild type RBD (Figure 2A; *p* < 0.001). At peak immunity following the second vaccination, there was a slight correlation in wild type Spike and RBD for both wild type and Omicron (Figure 2B, wild type *p* = 0.027, and Omicron *p* = 0.013), though this correlation plateaued at peak RBD levels. At six-months post mRNA vaccine, despite the fact that wild type RDB titers remained higher than Omicron RBD titers (Figure 2C; *p* < 0.01), there was a strong correlation in declining anti-wild type and Omicron RBD titers with anti-Spike titers (Figure 2D; wild type *p* = 0.0006, and Omicron *p* = 0.0007). These data underscore the need for long-term vaccine-induced pediatric immunoprotection amidst episodic surges of SARS-CoV-2 infections.

**Figure 3:**
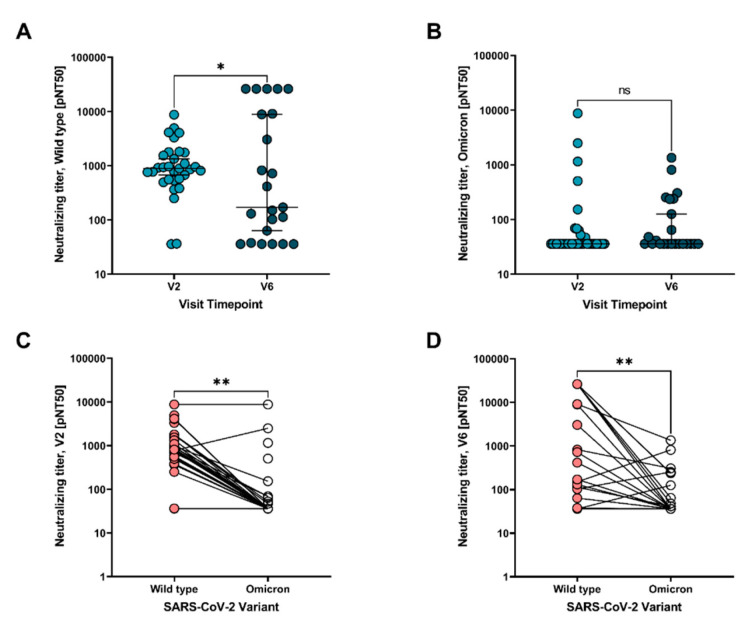
Comparison of neutralizing titers toward SARS-CoV-2 wild type and Omicron variant post mRNA vaccination. (**A**) Neutralizing titers measured following the second mRNA vaccine dose and 6-months post second vaccine dose toward SARS-CoV-2 wild type and (**B**) Omicron variant. Neutralizing titers measured against both SARS-CoV-2 wild type and Omicron variant at (**C**) V2 timepoint and (**D**) V6 timepoint. V2 = 2–4 weeks following the second mRNA vaccine dose, and V6 = 6 months following the second mRNA vaccine dose. IC50 = half maximal inhibitory concentration. Paired analysis with t-test. ns = not significant, * *p* < 0.05, ** *p* < 0.005. Median values and 95% CI are presented.

While total antibody quantification highlights humoral responses, assays testing the functional capacity of antibodies provide a deeper level of insight into immunoprotection. Viral neutralization and immune complex-mediated cellular activation against both wild type and Omicron SARS-CoV-2 variants were assessed following the second vaccine and again six months later. Neutralization against wild type SARS-CoV-2 declined significantly in most adolescents over time (Figure 3A; *p* = 0.01), although some individuals displayed an increase in neutralization by the six-month time point. No differences in neutralization capacity were seen at the V2 and V6 time points against Omicron (Figure 3B), and neutralization was significantly lower against Omicron than wild type at both time points (Figure 3C,D).

**Figure 4:**
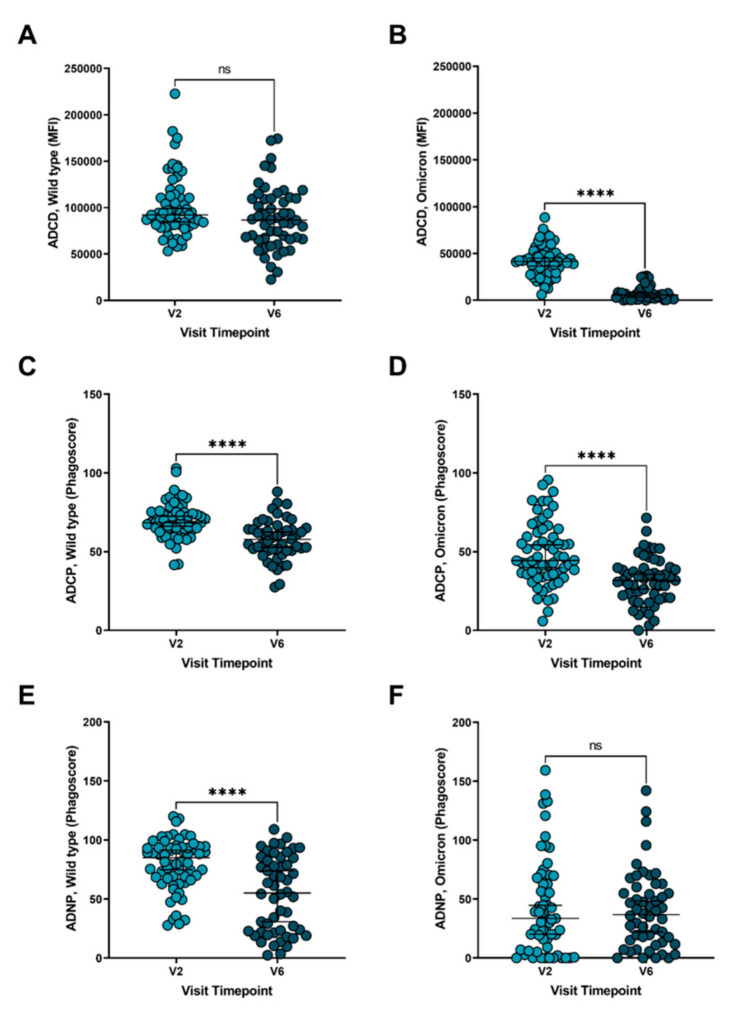
Comparison of humoral response titers toward SARS-CoV-2 wild type and Omicron variant post mRNA vaccination. Antibody-dependent complement deposition (ADCD) at the V2 and V6 timepoints against SARS-CoV-2 (**A**) wild type and (**B**) Omicron variant. Antibody-dependent cellular phagocytosis (ADCP) at the V2 and V6 timepoints against SARS-CoV-2 (**C**) wild type and (**D**) Omicron variant. Antibody-dependent neutrophil phagocytosis (ADNP) at the V2 and V6 timepoints against SARS-CoV-2 (**E**) wild type and (**F**) Omicron variant. V2 = 2–4 weeks following the second mRNA vaccine dose, and V6 = 6 months following the second mRNA vaccine dose. MFI = Mean Fluorescent Intensity. Paired analysis with t-test. ns = not significant, **** *p* < 0.0001. Median values and 95% CI are presented.

**Figure A2:**
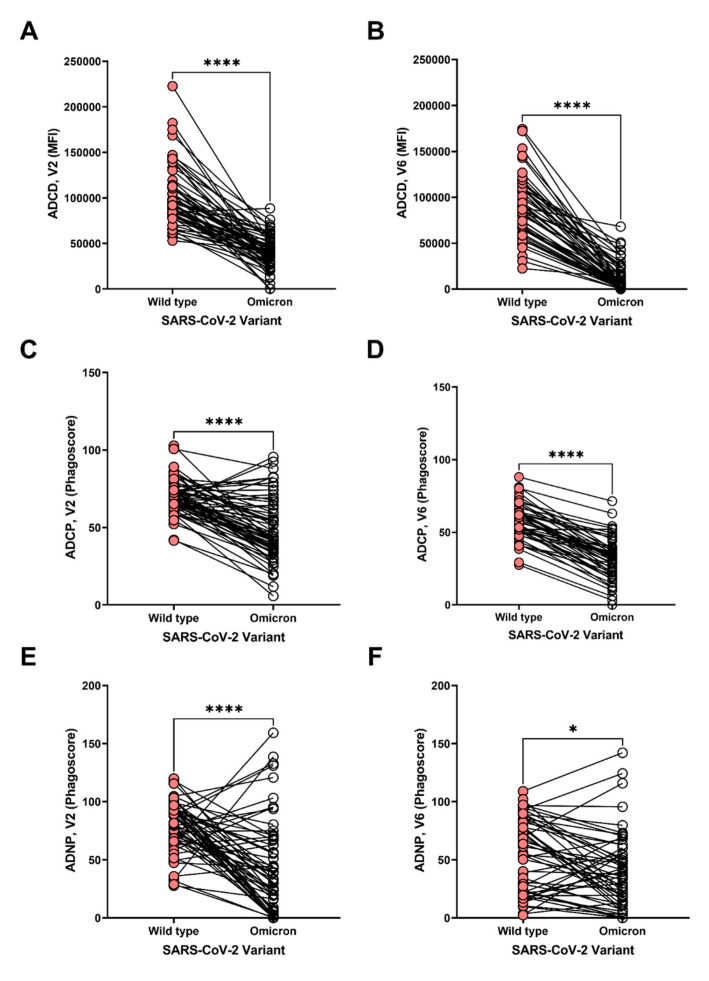
Comparison of humoral response titers at the V2 and V6 vaccine timepoint toward SARS-CoV-2 wild type and Omicron variant post-mRNA vaccination. (**A**) Antibody-dependent complement deposition (ADCD) at the V2 and (**B**) V6 timepoints against SARS-CoV-2 wild type and Omicron variant. (**C**) Antibody-dependent cellular phagocytosis (ADCP) at the V2 and (**D**) V6 timepoints against SARS-CoV-2 wild type and Omicron variant. (**E**) Antibody-dependent neutrophil phagocytosis (ADNP) at the V2 and (**F**) V6 timepoints against SARS-CoV-2 wild type and Omicron variant. V2 = 2–4 weeks following the second mRNA vaccine dose, and V6 = 6 months following the second mRNA vaccine dose. MFI = Mean Fluorescent Intensity. Paired analysis with t-test. * *p* < 0.05, **** *p* < 0.0001.

Enhancement of antibody responses by immune complex-mediated cellular activation is a central component of the humoral response. Antibody-dependent complement deposition (ADCD) showed a sustained response at both the V2 and V6 timepoint in the wild type strain (Figure 4A) but showed a significant decrease in the Omicron variant between the two timepoints (Figure 4B; *p* < 0.0001). Antibody-dependent cellular (THP-1 monocyte) phagocytosis (ADCP) displayed a significant decrease from the V2 to V6 time point in both wild type (Figure 4C; *p* < 0.0001) and Omicron strains (Figure 4D; *p* < 0.0001). Interestingly, while a pronounced decline in antibody-dependent neutrophil phagocytosis (ADNP) was seen between peak immunity and the six-month post-vaccine time point for the wild type strain (Figure 4E; *p* < 0.0001), neutrophil phagocytosis was sustained, albeit at a lower level, in the Omicron strain (Figure 4F; Figure A2A,B). Immune complex-mediated cellular activation was decreased overall for Omicron compared to wild type SARS-CoV-2 (Figure A2C–F).

## 4. Discussion

As the COVID-19 mRNA vaccines represent a new vaccination platform, the longevity of immune responses needs to be characterized across all age ranges, especially in light of emerging variants. Here, we detail the durability of antibody titers and functional capacity of humoral immune responses to the SARS-CoV-2 mRNA vaccine in adolescent children, including responses against the highly infectious and the current predominant variant, Omicron. As expected, and as seen in adult populations, mRNA vaccine-induced immunity in adolescent children wanes significantly over a 6-month time period, with a loss of circulating antibody titers and a reduction in antibody function, including viral neutralization. This finding demonstrates a current vulnerability to infection in adolescent children, many of whom have now received their vaccine series over six months ago.

While total anti-SARS-CoV-2 antibody titers and most anti-SARS-CoV-2 antibody functions waned over time in this adolescent cohort, immune complex-induced complement activation against wild type Spike persisted over time. Complement activation augments humoral responses by enhancing antibody neutralization of SARS-CoV-2 and promoting phagocytosis by immune cells [17]. This finding suggests that there may be lasting functional benefit of the antibodies even after only two mRNA vaccinations.

Encouragingly, our data demonstrate that adolescents’ immune responses display some cross-coverage of the VOC, Omicron, with comparable immune responses to those reported in adults [18]. This could suggest that adolescents develop a similar adaptive humoral immune response as seen in adults [19], which may provide benefits to children as additional variants of concern emerge. While it is plausible that vaccine-induced anamnestic immunity may provide some level of protection in this population upon exposure, the durability of vaccine-induced immunity wanes in adolescents, and boosting may promote a robust barrier of immunity, as previously reported in adults [20], that will contribute to public health efforts to limit spread and prevent future hospitalizations and severe disease. While cross-reactive immune complex-mediated activation was seen against Omicron, it was not as robust as wild type and waned more significantly over time, suggesting that functional aspects of cross reactivity benefits may be more apt to wane over time.

Our data support new CDC guidelines that recommend a booster dose five months from the completion of mRNA vaccination series to mitigate waning immunity [21]. Although three doses of the mRNA vaccine were suggested to have less protection against Omicron than for wild type and Delta variants, boosters are effective in reducing symptomatic infection, hospitalization, and death [22], and boosters will likely play a key role in preventing severe COVID-19-related disease in children, including MIS-C. As such, an additional booster dose may be sufficient for protection toward infection of current and future VOCs, as opposed to waiting for the construction and production of variant-specific vaccines. Overall, our data suggests the immunological need for adolescents to receive a third dose of the mRNA vaccine.

## 5. Conclusions

In conclusion, adolescent children exhibit waning antibody immune responses six months post-mRNA vaccination. mRNA boosters will play a critical role in sustaining durable immune responses in adolescent children, while also reducing pediatric infection, severe illness, and transmission as we traverse the surges of the COVID-19 pandemic.

## Figures and Tables

**Table 1:** Demographics of adolescents enrolled and timing of assessments for serologic responses to COVID-19 mRNA vaccination.

Patient Characteristics	Cohort (n = 77)
Age at Enrollment, mean (SD)	14 (1.8)
Male, number (%)	41 (53)
Hispanic, number (%)	15 (19)
Race, number (%)	
White	53 (69)
Black	2 (3)
Asian	1 (1)
Other	16 (21)
Unknown	5 (6)
Time Since Dose 1 and V1 Draw, average days (SD)	18 (3)
Time Since Dose 2 and V2 Draw, average days (SD)	18 (5)
Time Since Dose 2 and V6 Draw, average months (SD)	6.7 (0.5)

## Data Availability

Not applicable.
